# Ursodeoxycholic acid inhibits the proliferation of colon cancer cells by regulating oxidative stress and cancer stem-like cell growth

**DOI:** 10.1371/journal.pone.0181183

**Published:** 2017-07-14

**Authors:** Eun-Kyung Kim, Jae Hee Cho, EuiJoo Kim, Yoon Jae Kim

**Affiliations:** 1 Division of Gastroenterology, Department of Internal medicine, Gachon University Gil Medical Center, Incheon, the Republic of Korea; 2 Gachon Medical Research Institute, Gachon University Gil Medical Center, Incheon, the Republic of Korea; University of South Alabama Mitchell Cancer Institute, UNITED STATES

## Abstract

**Introduction:**

The regulation of reactive oxygen species (ROS) exists as a therapeutic target for cancer treatments. Previous studies have shown that ursodeoxycholic acid (UDCA) suppresses the proliferation of colon cancer cells. The aim of this study was to evaluate the effect of UDCA upon the proliferation of colon cancer cells as a direct result of the regulation of ROS.

**Method:**

Colon cancer cell lines (HT29 and HCT116) were treated with UDCA. The total number of cells and the number of dead cells were determined using cell counters. A fluorescein isothiocyanate-bromodeoxyuridine flow kit was used to analyze cell cycle variations. Upon exposure to UDCA, the protein levels of p27, p21, CDK2, CDK4 and CDK6 were determined using western blotting, and qRT-PCR was used to determine levels of mRNA. We preformed dichlorofluorescindiacetate (DCF-DA) staining to detect alteration of intracellular ROS using fluorescence activated cell sorting (FACS). Colon cancer stem-like cell lines were generated by tumorsphere culture and treated with UDCA for seven days. The total number of tumorspheres was determined using microscopy.

**Results:**

We found that UDCA reduced the total number of colon cancer cells, but did not increase the number of dead cells. UDCA inhibited the G1/S and G2/M transition phases in colon cancer cells. UDCA induced expression of cell cycle inhibitors such as p27 and p21. However, it was determined that UDCA suppressed levels of CDK2, CDK4, and CDK6. UDCA regulated intracellular ROS generation in colon cancer cells, and induced activation of Erk1/2. Finally, UDCA inhibited formation of colon cancer stem-like cells.

**Conclusion:**

Our results indicate that UDCA suppresses proliferation through regulation of oxidative stress in colon cancer cells, as well as colon cancer stem-like cells.

## Introduction

Colorectal cancer (CRC) is the third-leading diagnosed cancer in males and second-leading diagnosed cancer among females. Diagnosis rates have gradually increased and can be attributed to changes in diet, environmental factors, and genetic susceptibility. Despite advances in screening and treatment, CRC remains a leading cause of cancer-related death. Similar to other solid tumors, the main treatment methods for colon cancer are radiotherapy, surgery, and chemotherapy. Recently, treatment with specific monoclonal antibodies was also applied to advance CRC. However, new drugs or drug targets are needed for better treatment.

Ursodeoxycholic acid (UDCA) is used for the prevention of gall bladder stones, and in the treatment of primary biliary cirrhosis (PBC). It is the one of drugs that is approved by the United States Food and Drug Administration (US FDA, [1–3]) for the treatment of PBC. It helps regulate cholesterol absorption during the break-up of micelles containing cholesterol. UDCA has also proven effective as a preventative agent for inflammatory bowel disease [4], and has been shown to inhibit *in vivo* tumorigenesis in chemically induced colitis models of cells treated with dextran sodium sulfate (DSS)or azoxymethane (AOM, [5–9]).

Previous studies have demonstrated that UDCA can inhibit the proliferation of cancer cells. Specifically, the inhibition of colonic epithelium cell proliferation by UDCA has been observed in both patient and animal models of colon carcinogenesis [5, 10, 11]. The use of UDCA was not associated with an elevated risk of colorectal cancer or dysplasia in adult IBD patients with PBC; however, in one study, UDCA was found to be a source of heterogeneity [12]. Others have shown that UDCA has the ability to regulate oxidative stress in various diseases, including cancer, beyond the mechanisms for biliary tract diseases [13–15]. The purpose of this study was to investigate the mechanism of action of UDCA underlying the regulation of cell proliferation in colon cancer by means of oxidative stress.

## Materials and methods

### Reagents and materials

UDCA was obtained from Sigma-Aldrich (St. Louis, MO, USA). 2',7'-dichlorofluorescein diacetate (H2DCF-DA) was purchased from Molecular Probes (Eugene, OR, USA). Antibodies of phospho-Erk (#4370), total Erk (#4695), phospho-NF-κB p65 (#3033), total NF-κB p65 (#8242), phospho-p38 (#4511), total p38 (#8690), and cell cycle regulation sampler kit (#9932) were purchased from Cell Signaling Technology (Beverly, MA, USA). Anti-beta actin (LF-PA0207) was obtained from Ab Frontier (Seoul, Korea).

### Cell culture and treatment

Colon cancer HT29 and HCT116 cells were purchased from the Korean Cell Lines Bank (KCLB, Seoul, Korea) and cultured in McCoy’s medium (Gibco, NY, USA) supplemented with 10% fetal bovine serum (Gibco) and 1% antibiotic-antimycotic in a humidified 5% CO_2_ atmosphere. UDCA were diluted in DMSO. For experiments, 0.2mM of UDCA treated at 24 hr.

### Total cell counting

Cells were seeded and treated with UDCA for 24h, and then dissociated with trypsin-EDTA (Wellgene, Daegu, Korea) into single cell suspensions. The single cells were stained by trypan blue (Gibco) and counted using a Luna II^TM^ automated cell counter (Logos Biosystems, Anyang, Republic of Korea).

### Cell cycle analysis using flow cytometry

Cell cycle analysis was evaluated using a BD fluorescein isothiocyanate-bromodeoxyuridine (FITC-BrdU) flow kit (BD Pharmingen, San Diego, CA, USA). For BrdU incorporation, cells were incubated with 20 μM BrdU for 2 h before harvesting and then treated with 0.2 mM UDCA for 24 h. Cells were then washed with phosphate buffered saline (PBS) and fixed in Cytofix/Cytoperm^TM^ buffer. Fixed cells were treated with DNase for 1 hat 37°C and stained with FITC-conjugated anti-BrdU antibody for 20 min at room temperature. Finally, cells were stained with 7-aminoactinomycein (7-ADD) for 10min. Flow cytometry was performed using the BD FACS Calibur (BD Biosciences, Durham, NC, USA).

### Analysis of reactive oxygen species (ROS) by FACS

Intracellular ROS was assayed using H2DCF-DA. Cells were treated with 0.2 mM UDCA. After 20 min, cells were harvested by trypsin-ethylenediaminetetraacetic acid (EDTA) and washed with PBS. The harvested cells were incubated with 25μM H2DCFDA at 37°C for 30min. Fluorescence intensity was detected by flow cytometry.

### Immunoblotting

Collected cells were lysed in cold radioimmunoprecipitation assay (RIPA) lysis buffer (0.5 M Tris-HCl, pH 7.4, 1.5 M NaCl, 2.5% deoxycholic acid, 10% NP-40, 10 mM EDTA) with protease and phosphatase inhibitors (Gendepot, Katy, TX, USA). Cell lysates were subjected to sodium dodecyl sulfate polyacrylamide gel electrophoresis (SDS-PAGE) and transferred to a nitrocellulose membrane (Pall Gelman Laboratory, MI, USA). After blocking with 8% skim milk or 5% bovine serum albumin (BSA), the membrane was probed with primary antibodies overnight at 4°C. After washing with phosphate buffered saline/Tween-20 (PBST), the membrane was incubated with a secondary antibody conjugated to horseradish peroxidase viaa Western Bright ECL HRP substrate (Advansta, CA, USA).

### Reverse Transcription-PCR and quantitative reverse transcription PCR analyses

Total RNA was extracted from harvested cells using an RNeasy Plus Mini Kit (Qiagen, Hilden, Germany). The extracted total RNA was treated with DNase I (New England BioLabs).1μg of purified total RNA was reverse transcribed and amplified by RT-PCR using a High-Capacity cDNA Reverse Transcription Kit (Applied Biosystems, USA). For relative quantitation, reactions were tested using TaKaRa SYBR Premix Ex taq II (TaKaRa, Japan), and PCR processing was carried out in an iCycler (Bio-Rad, Hercules, CA, USA). The following primer pairs were used: human *p21*, 5'-TTAGCAGCGGAACAAGGAGT -3'and 5'-AGCCGAGAGAAAACAGTCCA -3'; human*p27*,5'-TAATTGGGGCTCCGGCTAACT -3' and 5'-TGCAGGTCGCTTCCTTATTCC -3'; human*CDK2*,5'-GTACCTCCCCTGGATGAAGAT -3' and 5'-CGAAATCCGCTTGTTAGGGTC -3'; human *CDK4*, 5'-CTGGTGTTTGAGCATGTAGACC -3' and 5'-GATCCTTGATCGTTTCGGCTG -3'; human *CDK6*, 5'-CCAGATGGCTCTAACCTCAGT -3' and 5'-AAC TTC CAC GAA AAA GAG GCT T-3'; *GAPDH*, 5'-AGG GCT GCT TTT AAC TCT GGT -3' and 5'-CCC CAC TTG ATT TTG GAG GGA -3'. Target genes were normalized to *GAPDH*. The fold change from untreated control was set at 1-fold, and the normalized fold change ratio was calculated.

### Floating-sphere formation assay

For the formation of colon cancer spheres, 4000 cells/well were seeded in six-well ultralow attachment plates (Corning Incorporated, Corning, NY, USA) in serum-free McCoy’s medium with B-27 supplement (50X, Gibco Invitrogen Corporation), 10ng/mL hFGF (R&D Systems, Minneapolis, MN, USA, #4114TC-01M), 10ng/mL hEGF (R&D, #236-EG-01M) and heparin (Sigma). Plating cells were incubated at 37°C in a 5% CO_2_atmosphere incubator. Following seeding, colon spheres were counted after 7 days.

### Statistical analysis

All data were analyzed by Student’s t test.

## Results

### Inhibition of cell proliferation by UDCA

**Fig 1:**
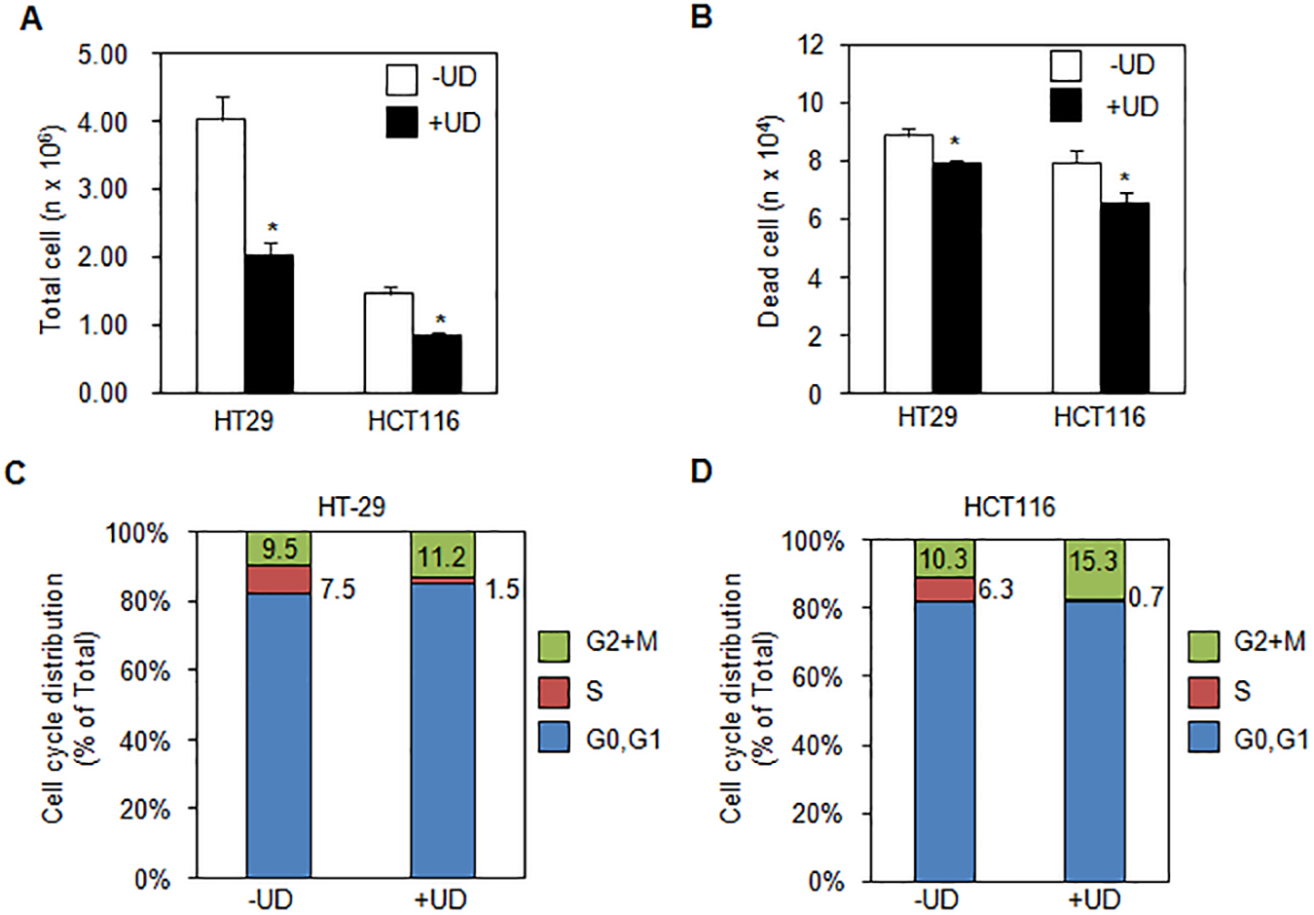
Ursodeoxycholic acid(UDCA)inhibited proliferation by cell cycle arrest in colon cancer cells. (A) Total cell number of HT29 or HCT116 reduced by treatment of 0.2 mM UDCA for 48h. (B) Dead cell counting of HT29 or HCT116 decreased by exposure to 0.2 mM UDCA at 48h. (C) S phase was decreased after treatment of 0.2 mM UDCA stained with BrdU and determined by flow cytometry. Asterisks indicate statistical significance (t<0.05).

To determine the anti-proliferative effect of UDCA, we counted the total number of HT29 and HCT116 cells using an auto cell counter (Fig 1A). As shown in the figure, the total number of HT29 cells diminished significantly following treatment with UDCA compared with untreated cells. The number of dead HT29 cells decreased as well, following treatment with UDCA (Fig 1B). Correspondingly, UDCA treatment led to a significant decrease in the total number of HCT116 cells and dead cells, alike.

To further investigate whether UDCA affects cell cycle progression, we utilized a FITC-BrdU Flow kit to examine the cell cycles of HT29 and HCT116 cells. A decrease in the population of G0/G1 and S phase was observed in UDCA-treated HT29 and HCT116 cells (Fig 1C). Furthermore, the total number of G2-M phase cells increased in both HT29 and HCT116 cell lines in the UDCA treatment group (Fig 1C and 1D). In the presence of UDCA, transitions from the G2/M phase to the G0/G1 phase and from G0/G1 to S phase were delayed in HT29 and HCT116. These results suggest that UDCA inhibits cell proliferation by alteration of cell cycle progression.

### UDCA-regulated the expression of cell cycle regulatory proteins

**Fig 2:**
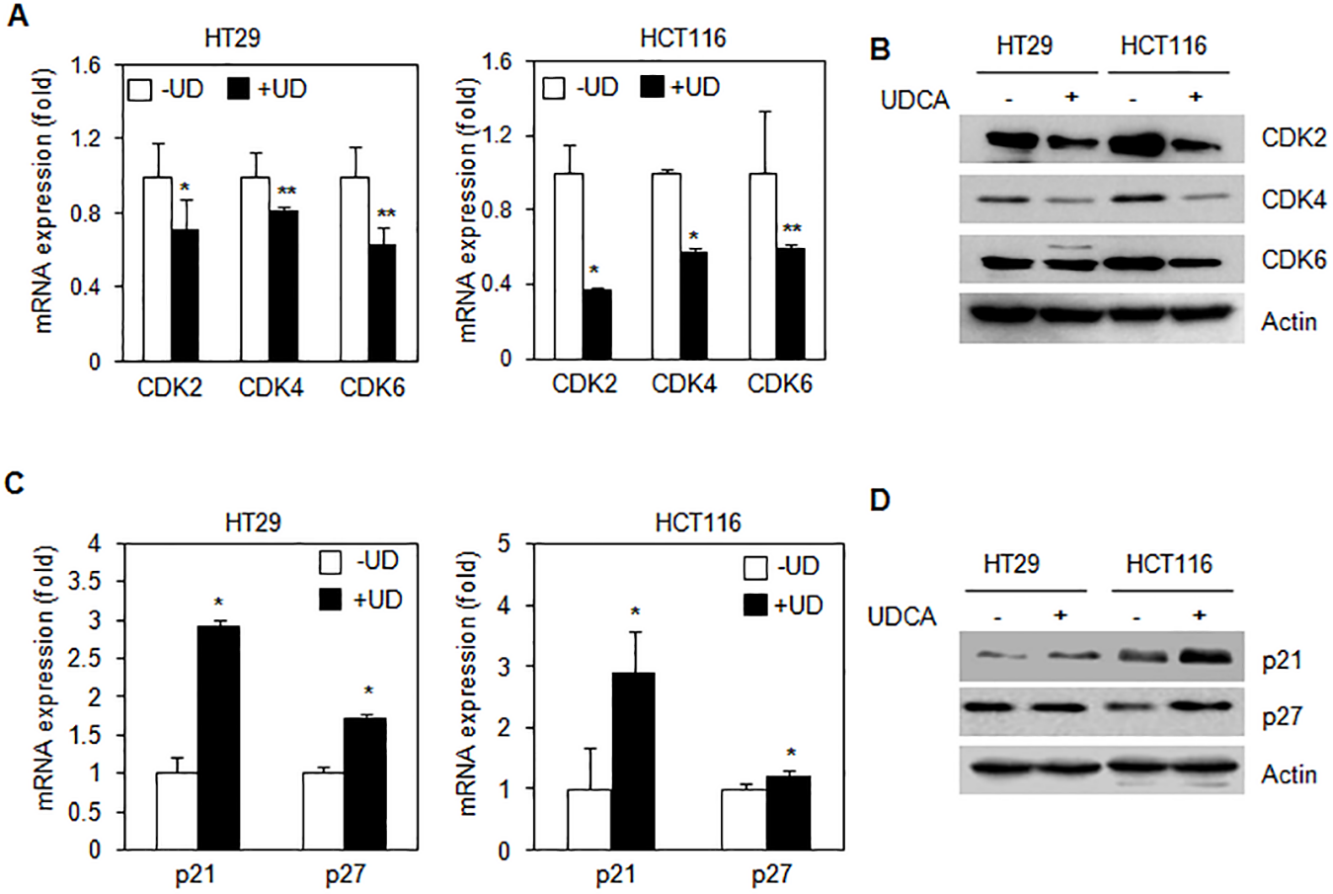
UDCA regulated levels of cell cycle related proteins. (A) mRNA expression of cell cycle related protein was quantified by qRT-PCR from HT29 and HCT116 cells with or without 0.2 mM UDCA for 24 h. (B) Decreased protein levels of cell cycle regulators in HT29 and HCT116 cells were observed over a 24h period by treatment with 0.2 mM UDCA. (C) mRNA levels of cell cycle inhibitors were shown by qRT-PCR from HT29 and HCT116 cells with and without treatment with 0.2 mM UDCA for 24 h. Error bars represent the standard deviation (SD); n = 3. (D) Protein levels of cell cycle inhibitors were evaluated by western blot from HT29 and HCT116 cells with or without treatment with 0.2 mM UDCA for 24 h. Experiments were performed in triplicate. Asterisks indicate statistical significance (t < 0.05).

To determine whether the presence of UDCA had an effect upon the expression of cell cycle regulators during cell proliferation, we investigated the production of mRNA and the protein levels of cyclin-dependent kinases. UDCA was found to inhibit the mRNA expression of CDK2, CDK4, and CDK6 in HT29 and HCT116 cells (Fig 2A). Furthermore, as depicted in the Fig 2B, UDCA suppressed the expression of CDK2, CDK4, and CDK6 proteins.

We then tested whether or not treatment with UDCA regulated control of cyclin-dependent kinase inhibitors such as p21, and p27. mRNA expression of p21 and p27 was increased following treatment with UDCA (Fig 2C). UDCA induced an increase in protein levels of p21, and p27 in HT29 and HCT116 cells, as shown in Fig 2D.

### UDCA-enhanced activation of Erk1/2 and p38 by reduction of intracellular ROS in colon cancer

**Fig 3:**
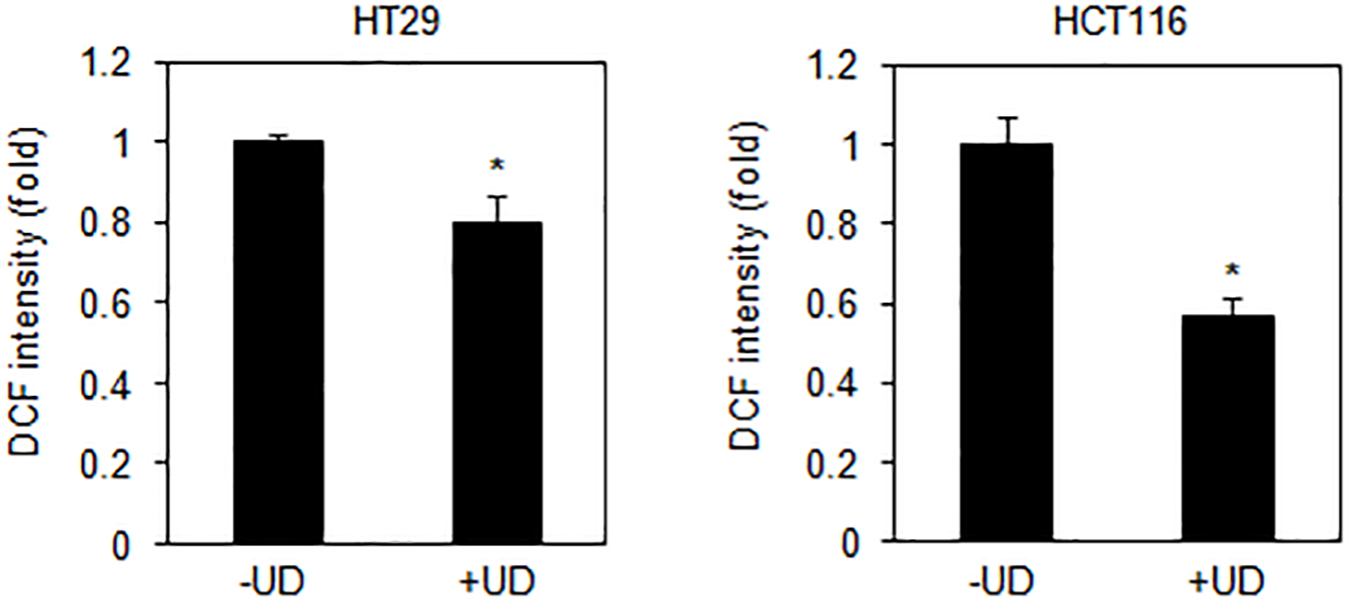
Effect of UDCA on removal of intracellular reactive oxygen species (ROS). HT29 and HCT116 cells were treated in 0.2 mM UDCA and stained with 25 μM H2DCF-DA for 30 min. Total levels of intracellular ROS were detected by fluorescence activated cell sorting (FACS) analysis. Asterisks indicate statistical significance (t<0.04).

In an attempt to evaluate the changes in intracellular ROS levels due to UDCA during proliferation, we investigated the antioxidant effect of UDCA upon colon cancer cell lines. The diminution of ROS by UDCA was detected using DCFDA staining and FACS. The levels of total ROS were reduced following UDCA treatment in both HT29 and HCT116 cell lines (Fig 3).

**Fig 4:**
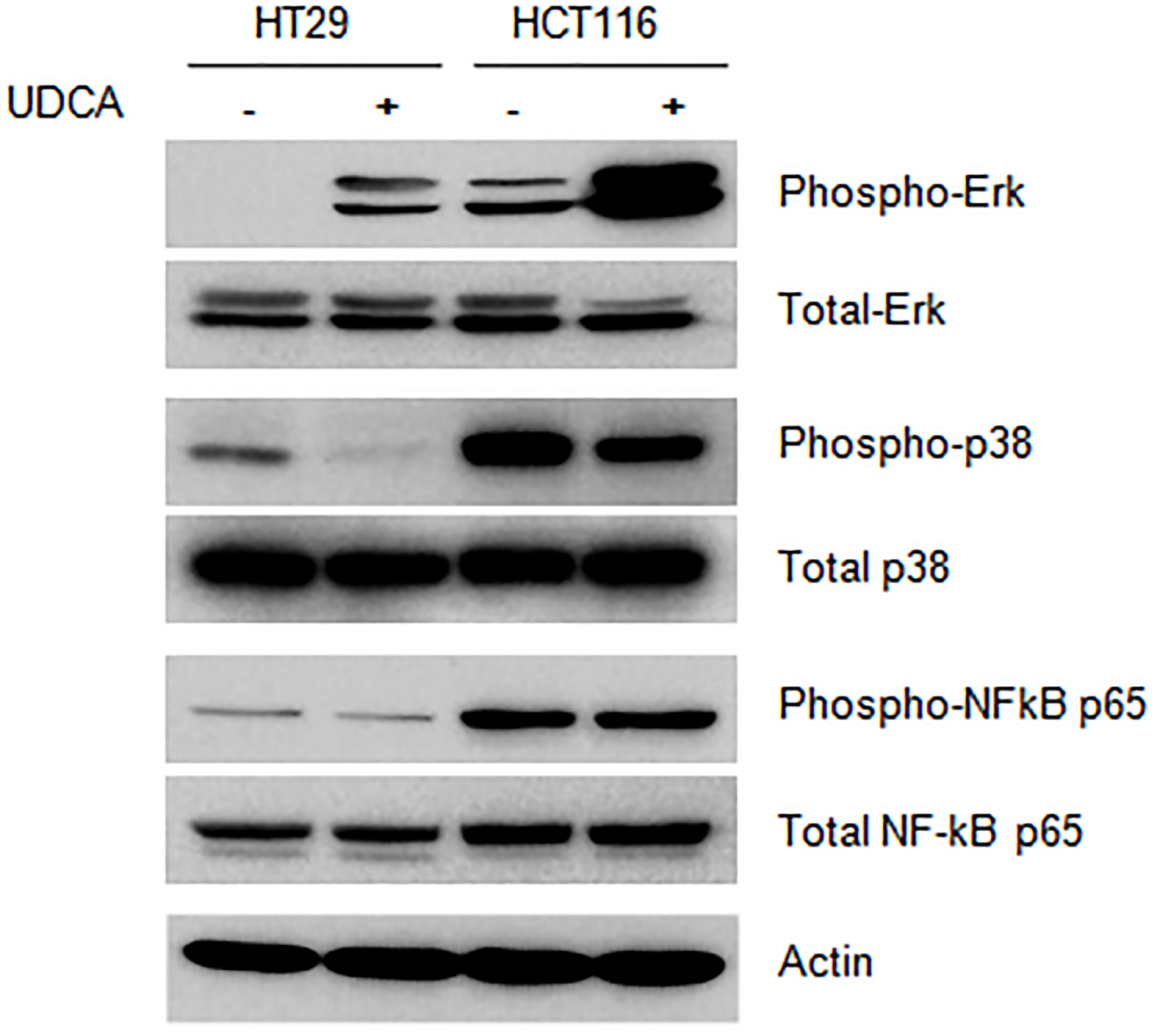
UDCA regulated activation of Erk and p38 pathways. HT29 and HCT116 cells were treated with 0.2 mM UDCA for 30 min. The data are representative of experiments performed in triplicate.

To clarify the mechanism of action of UDCA, the screening of pathway was performed by using phosphor-antibodies. Treatment with UDCA enhanced Erk1/2 phosphorylation in HT29 and HCT116 cells (Fig 4). Furthermore, treatment with UDCA decreased phosphorylation of p38 in HT29 and HCT116 cells. Phosphorylation of NF-κB p65 in the same cell lines remained unchanged following treatment with UDCA. This data suggests that UDCA may mediate inhibition of cell proliferation by regulation of Erk1/2 and p38 in HT29 and HCT116 cell lines.

### UDCA inhibited the formation of colon cancer stem-like cells

**Fig 5:**
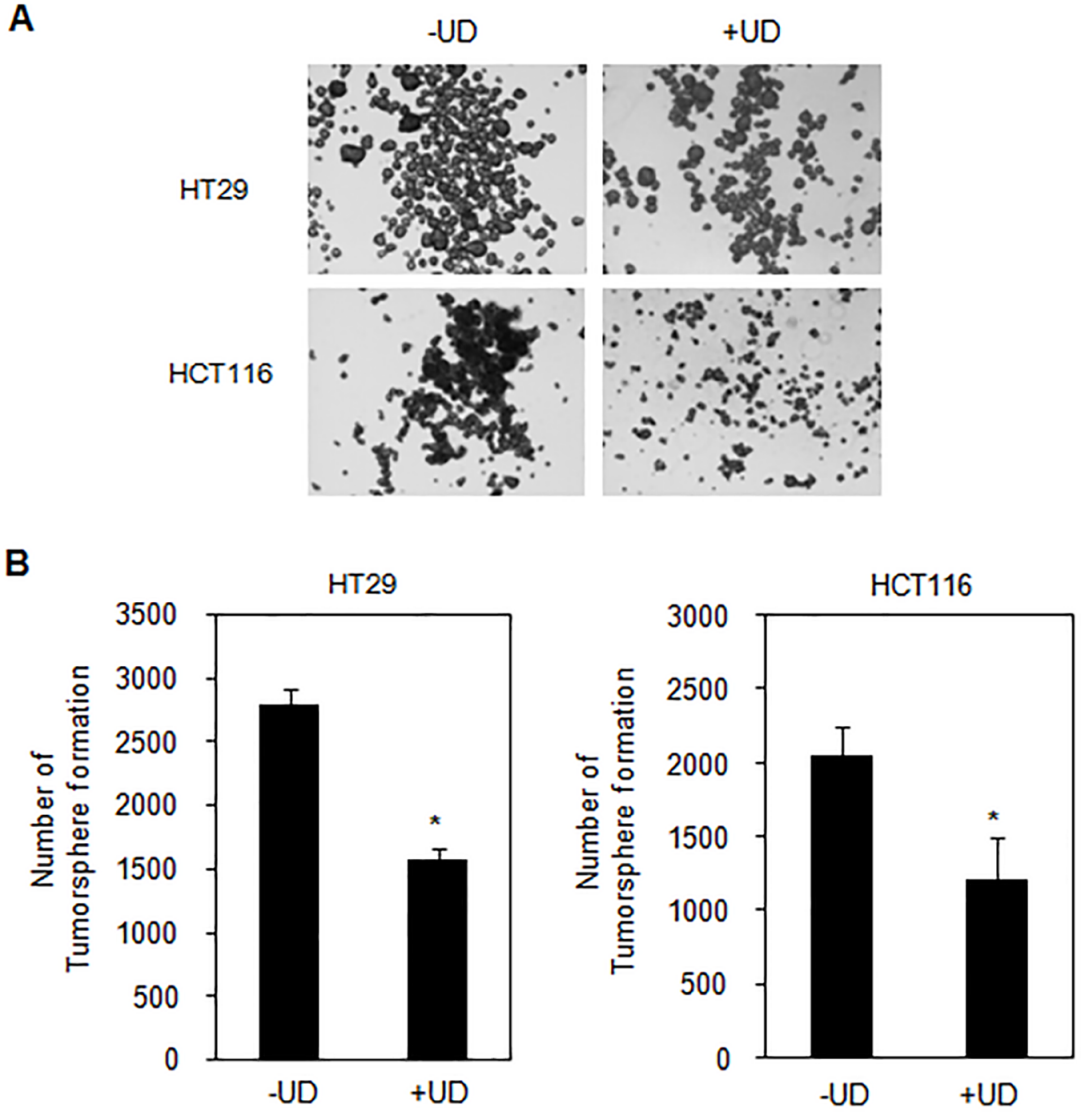
UDCA diminished the number of colon tumorspheres. (A) Representative images of untreated and 0.2mM UDCA-treated tumorspheres. Images were taken with a 4X objective. (B) Quantification of tumorsphere-forming potential in untreated spheres or in spheres treated with 0.2 mMUDCA for 7days. Error bars denote standard error (n = 3, t< 0.001).

In an attempt to investigate whether UDCA suppressed the formation of tumorspheres, we cultured HT29 and HCT116 cells in ultralow attachment plates in the presence and absence of UDCA for 7days. Fig 5A depicts the morphology of colon tumorspheres, which decreased in the presence of UDCA. Moreover, the formation of tumorspheres was reduced by UDCA in HT29 and HCT116 cells (Fig 5B). These results suggest that UDCA represses the formation of tumorspheres in colon cancer.

## Discussion

Several studies have suggested that UDCA has a chemo-preventive and anticancer effect on gastrointestinal tract cancers that extends beyond its role as a bile acid[16–18]. Wuang *et al*. reported that UDCA reduces the risk of colon cancer development in a cohort study on liver disease. Akare *et al*. reported that UDCA exhibits a chemo-preventive effect via modulation of chromatin by inducing hypoacetylation and differentiation of colon cancer cells. Histone deacetylase inhibitor (HDAC) 6 is upregulated by UDCA and plays a role in UDCA-induced senescence, although p53, p21 and Rb were not affected after UDCA treatment[18]. Several UDCA-mediated mechanisms that could potentially repress the growth of cancer cells have been identify, however, the inhibitory molecular mechanism of proliferation by UDCA has not been completely elucidated.

Herein, we report two important mechanisms of action for UDCA on colon cancer cells: cell cycle regulation and reduction of intracellular ROS. We showed UDCA-mediated inhibition of proliferation in HT29 and HCT116 cells; however, there was no observable increase in number of dead cells. After 24 h of treatment with 0.2 mM UDCA, the total cell count was reduced in HT29 and HCT116 cells (Fig 1A and 1B). These effects result from repression of cell cycle progression. Specifically, UDCA induced cell cycle arrest from G2/M phase to S phase (Fig 1C and 1D). The inhibition of proliferation of HCT117 and HT29 colon carcinoma cell lines within 24h has been previously associated with the detection of G1 and G2/M cell arrest[18–20]. This inhibitory effect was also observed in patients with colorectal neoplasia upon treatment with UDCA [21].

This study reveals that UDCA is capable of regulating intracellular ROS levels by acting as a scavenger in colon cancer cell lines (Fig 2). Reduction of ROS levels by UDCA may influence the activity of cellular signaling molecules during cell proliferation and cell cycle progression. Regulation of secondary messengers, such as p21 and MAPK, are influenced by ROS and can subsequently affect proliferation[22]. *N*-acetyl-L-cysteine [23] is a known ROS inhibitor that can induce cell cycle arrest at the G1 phase via regulation of Erk1/2 phosphorylation and p21 expression [24]. In other reports, α-lipoic acid, an antioxidant, induced p27-mediated cell cycle arrest in human breast cancer cells [25].

Phosphorylation of Erk1/2 not only induces G1-S phase transition [26–28], but also mediates the inhibition of cell growth [29–31]. Fig 3 shows the increase in phosphorylation that was observed for Erk1/2 as a result of treatment with UDCA. Erk1/2 activation-induced arrest of cell cycle progression in colon carcinoma and colonic epithelial cells is well known [26, 32, 33]. p38 kinase is also known to contribute to cellular proliferation and stress response. Fig 3 shows that inactivation of p38 kinase by UDCA suppressed proliferation. Thus, UDCA may regulate the activity of Erk1/2 and p38 by modulation of phosphorylation states between Erk1/2 and p38 kinases.

UDCA-mediated cell cycle inhibition was induced by regulation of the expression of cell cycle-related proteins. Activation of the cell cycle is induced by the cyclin dependent kinases CDK2, CDK4, and CDK6. The expression of these proteins was affected by UDCA (Fig 4A and 4B), although it does not seem to affect protein stability. Instead, UDCA appears to inhibit mRNA expression of these proteins. UDCA is not a transcription factor, but it has been shown to affect transcription factors such as NF-κB, AP-1, and p53 [34–39]. UDCA altered the capacity for transcription by these transcription factors perhaps by having an effect upon DNA binding. Further, UDCA positively regulates mRNA expression of p21 and p27, two CDK inhibitors (Fig 4C and 4D). Previous studies revealed that cells treated with UDCA induced the inhibition of CDK levels and suppressed cellular proliferation in HCT8 cells [40].

We also investigated the inhibitory role of UDCA on cell proliferation and its affect upon cancer stem-like cells. To evaluate the viability of stem-like cells, cancer cells were cultured on ultralow-attachment plates to form tumorspheres [41], [42]. We have shown that UDCA reduced the formation of tumorspheres in HT29 and HCT116 cells (Fig 5). These results suggest that UDCA inhibits the formation of cancer stem cells, and that UDCA may serve as an effective supplemental medicine for reducing the growth of cancer stem cells.

Our novelty of investigation reveals that UDCA works to activate phosphorylation of p38 and hyper-phosphorylation of Erk1/2 by repression of ROS generation and then, regulates expression of cell cycle related protein in progression of cell proliferation.

There have been several molecules shown to possess chemo-preventive and anticancer effects in colon cancer. The results presented herein demonstrate that the mechanism of action of UDCA likely involves regulation of the cell cycle through reduction of ROS. UDCA also appears to be a viable inhibitor of cancer stem-cell formation. Further clinical studies of UDCA on colon cancer patients are needed to evaluate the clinical viability of UDCA.
